# The Risk of Chronic Gastrointestinal Disorders Following Acute Infection with Intestinal Parasites

**DOI:** 10.3389/fmicb.2018.00017

**Published:** 2018-01-23

**Authors:** Jason Blitz, Mark S. Riddle, Chad K. Porter

**Affiliations:** ^1^Navy Environmental and Preventive Medicine Unit Six, Pearl Harbor, HI, United States; ^2^Department of Preventive and Biostatistics, Uniformed Services University of the Health Sciences, Bethesda, MD, United States; ^3^Naval Medical Research Center, Silver Spring, MD, United States

**Keywords:** protozoans, functional bowel disorder, epidemiology, parasites, sequela

## Abstract

**Background:** Infectious gastroenteritis (IGE) is caused by numerous bacterial, viral, and parasitic pathogens. A history of IGE has been shown in previous studies to increase the risk of developing chronic gastrointestinal disorders and other chronic conditions. As bacteria and viruses represent the majority of pathogen-specific causes of IGE, post-infectious studies have primarily focused on these organisms. The objective of this study was to investigate an association between a history of parasite-associated IGE and the subsequent development of chronic post-infectious gastrointestinal and non-gastrointestinal disorders in a military population.

**Methods:** International Classification of Diseases, 9th Revision Clinical Modification (ICD-9-CM) diagnostic coding data for primary exposures and outcomes were obtained for a retrospective cohort study of active component military personnel from 1998 to 2013. Exposed subjects consisted of individuals with documented infection with one of ten parasitic pathogens. Unexposed subjects were matched to exposed subjects on demographic and operational deployment history parameters. Adjusted odds ratios (aORs) were estimated using logistic regression for several chronic disorders previously shown to be associated with a history of IGE.

**Results:** A total of 896 subjects with a parasitic exposure were matched to 3681 unexposed subjects for multivariate regression analysis. Individuals infected with *Balantidium coli*, *Ascaris lumbricoides*, *Strongyloides stercoralis*, *Necator americanus/Ancylostoma duodenale*, and *Taenia* spp. had higher aOR for development of several chronic gastrointestinal disorders when compared with unexposed subjects after controlling for various covariates.

**Conclusion:** We found that parasite-associated enteric infection increases the risk of development of post-infectious chronic gastrointestinal disorders in a military population. These results require confirmation in similar populations and in the developing world where infection with these parasites is endemic. Further understanding of disease burden and causal mechanisms should direct primary prevention and potential disease interception strategies.

## Introduction

A growing body of literature has linked enteric infection with an increased risk of a number of chronic gastrointestinal (GI) disorders including irritable bowel syndrome (IBS), gastroesophageal reflux disease (GERD), and non-GI disorders such as chronic fatigue syndrome (CFS) (Connor and Riddle, 2013; Hanevik et al., 2014; Futagami et al., 2015; Schwille-Kiuntke et al., 2015b). Proposed mechanisms include functional changes such enterochromaffin cell hyperplasia, altered serotonin synthesis and reuptake, impaired gut mucosal barrier function, altered immune activation, and dysfunctional mast cell activation (Verdu and Riddle, 2012; Deising et al., 2013).

To date, the study of intestinal parasites and chronic GI disorders has been limited predominately to *Giardia lamblia.* Specifically, in the 12–30 months following a waterborne disease outbreak due to an acute Giardia infection, 80.5% of infected subjects had IBS symptoms and 24.3% had functional dyspepsia (Hanevik et al., 2009). Follow-up studies in this cohort have demonstrated a persistence in post-infectious IBS symptoms as well as an increase in the risk of chronic fatigue (Naess et al., 2012; Wensaas et al., 2012; Hanevik et al., 2014). The risk of chronic GI outcomes has since been reported in a different population following sporadic Giardia infections (Dormond et al., 2016). Other enteric parasites, although less common than giardia, may be similarly associated with chronic sequelae. To that end, we sought to assess the risk of several post-infectious outcomes after an acute infection with one of the following parasites *Balantidium coli*, *Strongyloides stercoralis, Ascaris lumbricoides, Necator americanus, Ancylostoma duodenale, Taenia solium, Taenia saginata,* and *Hymenolepis nana.* While many of these infections are asymptomatic, it is theorized that infection with such organisms could trigger changes in immune function, metabolic processes, and intestinal microflora populations and result in abnormal intestinal or extraintestinal physiology that could be associated with symptoms persisting well after the infection has cleared (Fonseca et al., 2015).

## Materials and Methods

We utilized a retrospective cohort study to estimate the risk of post-infectious sequelae following a documented episode of parasite-associated enteric infection compared to the risk of those same outcomes in a cohort of subjects without a parasitic enteric infection. Subjects with enteric parasites were identified from the Defense Medical Surveillance System (DMSS) from 1998 to 2013 using the International Classification of Diseases, Volume 9 – Clinical Modification (ICD-9-CM) diagnostic coding data. Each exposed subject was matched to four unexposed subjects with an ICD-9-CM medical encounter unrelated to IGE (acute upper respiratory infection, sprains, strains, and dislocations). Matching was based on age (within 1 year), gender, military treatment facility, type of medical encounter (inpatient/outpatient), time of identifying medical encounter or reportable event (within 1 year), and number of prior operational deployments. All subjects had a minimum of 1 year of documented follow-up within the DMSS.

Data were supplied by the Defense Health Agency – Armed Forces Health Surveillance Branch (DHA-AFHSB) which oversees the DMSS and is a main data repository for all medical encounters for United States military personnel including demographic and operational deployment history. The DMSS includes ambulatory data from the Standard Ambulatory Data Record (SADR) and Comprehensive Ambulatory Provider Encounter Record (CAPER), inpatient data from the Standard Inpatient Data Record (SIDR), and medical encounters recorded by the Tri-Service Reportable Events System. Identifiable information was removed from the datasets by DHA-AFHSB prior to being released to the investigators.

The primary exposure variable was enteric parasite infection attributed to one of the following parasitic pathogens (ICD-9-CM code): *Balantidium coli* (007.0), *Strongyloides stercoralis* (127.2)*, Ascaris lumbricoides* (127.0)*, Necator americanus* (126.9)*, Ancylostoma duodenale* (126.1)*, Taenia solium* (123.0)*, Taenia saginata* (123.2), and *Hymenolepis nana* (123.6). Subjects were chosen from patients with one or more medical encounters with ICD-9-CM codes associated with the primary exposure in the first diagnostic position or from the Tri-Service Reportable Events database. The outcomes of interest (and ICD-9-CM codes) were IBS (564.1, 306.4), dyspepsia (536.8), functional constipation (564.0x), functional diarrhea (564.5), and GERD (530.81). All subjects had a negative medical history for the outcomes of interest prior to enrollment into the cohort.

Psychological co-morbidities have been shown to increase a person’s risk for many FGIDs and other chronic conditions, therefore data for specific psychiatric disorders were obtained for all subjects (Drossman, 2006; Longstreth et al., 2006; Thabane et al., 2007; Porter et al., 2011a). Additionally, an increased risk of the development of PI sequelae has been shown to be associated with a previous IGE-related medical encounter (Porter et al., 2011b). Therefore, exposure to bacterial, viral, and parasitic pathogens (in addition to the specific parasites of interest) was assessed using ICD-9-CM codes. Other potential confounders such as age, race, highest education level, military branch, military rank, and number of previous operational deployments were also obtained.

Continuous variables were analyzed using a Student’s *t*-test and categorical variables by a Cochran Chi-square test. Univariate and multivariate modeling was conducted using logistic regression. Using a backward elimination approach to avoid under fitting, all variables were initially included in the multivariate model and removed sequentially for an alpha >0.20. Statistical analysis was conducted using SAS 9.3 (SAS Institute, Raleigh, NC, United States). The study protocol was approved as an Exempt research study by the Naval Medical Research Center Institutional Review Board in compliance with all applicable Federal regulations governing the protection of human subjects. The Exempt designation was based on the fact that the research involved the use of existing data in which subjects could not be identified, directly or through identifiers linked to the subjects.

## Results

A total of 896 exposed subjects and 3,681 matched unexposed subjects were identified (**Table 1**). A majority of subjects were male (88.6%), were white (62.5%), with a high school level of education or equivalent (68.2%), junior enlisted rank (51.7%), and with a mean age of 28.0 (SD: 7.6) years. A little more than a quarter (29.9%) of subjects had a history of deployment prior to enrollment in the cohort. Service distribution was roughly equivalent to the general active component military population with the highest proportion in the Army (32.8%) and fewest in the Coast Guard (5.0%).

**Table 1:** Demographic characteristics of United States active component study subjects from 1998 to 2013 by primary exposure of interest.

	Parasitic exposure of interest								
	*B. coli*	*S.*	*A.*	*N. americanus*	*T. solium*	*H. nana*	Not exposed	All exposed	Total
		*stercoralis*	*lumbricoides*	and *A. duodenale*	and *T. saginata*				
*N*	339	15	124	155	240	23	3681	896	4577
Mean (*SD*) age	26.4 (7.0)	33.1 (11.2)	28.2 (7.6)	29.3 (7.6)	29.3 (7.9)	29.0 (6.4)	28.0 (7.6)	28.1 (7.7)	28.0 (7.6)
Gender; *n* (%)					
Male	338 (99.7)	15 (100.0)	91 (73.4)	137 (88.4)	211 (87.9)	19 (82.6)	3246 (88.2)	811 (90.5)	4057 (88.6)
Female	1 (0.3)	0 (0.0)	33 (26.6)	18 (11.6)	29 (12.1)	4 (17.4)	435 (11.8)	85 (9.5)	520 (11.4)
Race; *n* (%)					
White	147 (43.4)	7 (46.7)	74 (59.7)	121 (78.1)	130 (54.2)	13 (56.5)	2367 (64.3)	492 (54.9)	2859 (62.5)
Black	81 (23.9)	2 (13.3)	17 (13.7)	5 (3.3)	65 (27.1)	6 (26.1)	593 (16.1)	176 (19.6)	769 (16.8)
Other	111 (32.7)	6 (40.0)	33 (26.6)	29 (18.7)	45 (18.8)	4 (17.4)	721 (19.6)	228 (25.4)	949 (20.7)
Education; *n* (%)					
High school (or equivalent)	272 (80.2)	7 (46.7)	80 (64.5)	79 (51.0)	147 (61.3)	14 (60.9)	2521 (68.5)	599 (66.9)	3120 (68.2)
Some college	26 (7.7)	1 (6.7)	17 (13.7)	29 (18.7)	43 (17.9)	5 (21.7)	456 (12.4)	121 (13.5)	577 (12.6)
College	19 (5.6)	3 (20.0)	17 (13.7)	32 (20.7)	22 (9.2)	2 (8.7)	389 (10.6)	95 (10.6)	484 (10.6)
Graduate school	11 (3.2)	2 (13.3)	8 (6.5)	9 (5.8)	20 (8.3)	1 (4.4)	173 (4.7)	51 (5.7)	224 (4.9)
Unknown	11 (1.2)	2 (13.3)	2 (1.6)	6 (3.9)	8 (3.3)	1 (4.4)	142 (3.9)	30 (3.4)	172 (3.8)
Rank; *n* (%)					
Jr. enlisted	198 (58.8)	5 (33.3)	64 (52.0)	58 (38.7)	108 (45.2)	9 (39.1)	1898 (52.1)	442 (49.8)	2340 (51.7)
Sr. enlisted	21 (6.2)	4 (26.7)	25 (20.3)	0 (0.0)	29 (12.1)	0 (0.0)	1225 (33.6)	323 (36.4)	1548 (34.2)
Jr. officer	116 (34.4)	5 (33.3)	33 (26.8)	36 (24.0)	101 (42.3)	2 (8.7)	485 (13.3)	117 (13.2)	602 (13.3)
Sr. officer	2 (0.6)	1 (6.7)	1 (0.8)	56 (37.3)	1 (0.4)	12 (52.2)	34 (0.9)	5 (0.6)	39 (0.9)
Service; *n* (%)					
Army	101 (29.8)	6 (40.0)	47 (37.9)	38 (24.5)	76 (31.7)	9 (39.1)	1223 (33.2)	277 (30.9)	1500 (32.8)
Navy	67 (19.8)	5 (33.3)	26 (21.0)	27 (17.4)	56 (23.3)	3 (13.0)	775 (21.1)	184 (20.5)	959 (21.0)
Air force	111 (32.7)	0 (0.0)	38 (30.7)	43 (27.7)	70 (29.2)	8 (34.8)	1035 (28.12)	270 (30.1)	1305 (28.5)
Marines	50 (14.8)	4 (26.7)	10 (8.1)	16 (10.3)	28 (11.7)	2 (8.7)	476 (12.9)	110 (12.3)	586 (12.8)
Coast guard	10 (3.0)	0 (0.0)	3 (2.4)	31 (20.0)	10 (4.2)	1 (4.4)	172 (4.7)	55 (6.1)	227 (5.0)
Operational deployment^∗^; *n* (%)	110 (32.5)	4 (26.7)	39 (31.5)	53 (34.2)	59 (24.6)	12 (52.2)	1090 (29.6)	277 (30.9)	1367 (29.9)

^∗^*Limited to deployments prior at the time of enrollment into the cohort*.

*Balantidium coli* was the most common infection (*n* = 339; 37.8%) followed by *Taenia* spp. (*n* = 240; 26.8%), *N. americanus*/*A. duodenale* (*n* = 155; 17.3%), and *A. lumbricoides* (*n* = 124; 13.8%). Only 23 exposures to *H. nana* and 15 to *S. stercoralis* were identified which limited multivariate estimates for these infections.

In univariate and multivariate analyses (**Table 2**), infection with any one of the included parasites was associated with a statistically significant increased risk of functional constipation (OR: 4.13; aOR: 4.18), functional dyspepsia (OR: 3.30; aOR: 3.25), and GERD (OR: 2.14; aOR: 2.13) as well as a non-significant increased risk of IBS (OR: 1.69; aOR: 1.76).

**Table 2:** Unadjusted and adjusted odds ratios (aORs) and 95% Wald confidence intervals for irritable bowel syndrome (IBS), functional dyspepsia, functional constipation, and gastroesophageal reflux disease (GERD).

Risk factor	IBS		Functional dyspepsia		Functional constipation		GERD	
	OR (95% CI)	aOR (95% CI)	OR (95% CI)	aOR (95% CI)	OR (95% CI)	aOR (95% CI)	OR (95% CI)	aOR (95% CI)
Parasite (any)	1.69 (0.77, 3.68)	1.76 (0.81, 3.85)	3.30 (1.80, 6.05)	3.25 (1.77, 5.98)	4.13 (1.33, 12.83)	4.18 (1.34, 13.07)	2.14 (1.51, 3.03)	2.13 (1.50, 3.03)
Female	3.23 (1.48, 7.05)	3.31 (1.52, 7.24)	1.80 (0.83, 3.89)	1.94 (0.89, 4.24)	2.61 (0.70, 9.67)	3.32 (0.88, 12.48)	1.29 (0.81, 2.07)	1.45 (0.90, 2.34)
Race					
White	Reference		Reference		Reference		Reference	
Black	0.65 (0.22, 1.87)	–	1.53 (0.70, 3.33)	–	1.24 (0.25, 6.16)	–	1.41 (0.92, 2.14)	–
Other	0.52 (0.18, 1.51)		1.65 (0.81, 3.35)		2.01 (0.57, 7.15)		1.28 (0.86, 1.92)	
Branch of service				–		–		
Army	Reference		Reference		Reference		Reference	
Coast guard	1.53 (0.43, 5.42)	–	1.20 (0.67, 5.46)		1.32 (0.15, 11.37)		1.08 (0.51, 2.32)	–
Air force	1.06 (0.48, 2.34)		1.89 (0.89, 4.02)		0.69 (0.16, 2.89)		1.03 (0.68, 1.56)	
Marines	0.00 (UAE^2^)		0.70 (0.19, 2.51)		0.51 (0.06, 4.38)		0.62 (0.33, 1.17)	
Navy	0.36 (0.10, 1.26)		1.28 (0.53, 3.11)		0.63 (0.12, 3.23)		1.16 (0.75, 1.79)	
Rank					
Jr. enlisted	Reference		Reference	Reference	Reference	Reference	Reference	Reference
Sr. enlisted	1.11 (0.51, 2.42)	–	1.52 (0.81, 2.83)	1.34 (0.70, 2.56)	4.05 (1.07, 15.28)	4.23 (1.12, 16.10)	1.93 (1.35, 2.76)	1.87 (1.30, 2.69)
Officer	1.13 (0.41, 3.13)		0.51 (0.15, 1.71)	0.32 (0.09, 1.21)	1.13 (0.12, 10.90)	1.13 (0.12, 10.90)	1.41 (0.86, 2.31)	1.39 (0.85, 2.28)
>High school education	1.83 (0.86, 3.88)	–	1.44 (0.78, 2.69)	1.92 (0.97, 3.80)	1.22 (0.37, 4.04)	–	1.16 (0.81, 1.66)	
IGE^1^	1.70 (0.52, 5.64)	–	1.22 (0.37, 3.96)	–	0.00 (UAE^2^)	–	1.36 (0.72, 2.54)	
Psych dx^1^	1.40 (0.66, 2.98)	–	1.57 (0.84, 2.97)	–	2.09 (0.66, 6.60)	–	1.14 (0.79, 1.65)	
Operational deployment^1^	0.82 (0.40, 1.66)	–	0.97 (0.53, 1.78)	–	1.08 (0.34, 3.40)	–	1.36 (0.97, 1.91)	1.28 (0.91, 1.80)

^1^*Limited to medical encounters prior to meeting the endpoint of interest; ^2^unable to estimate due to 0 cell*.

**FIGURE 1:**
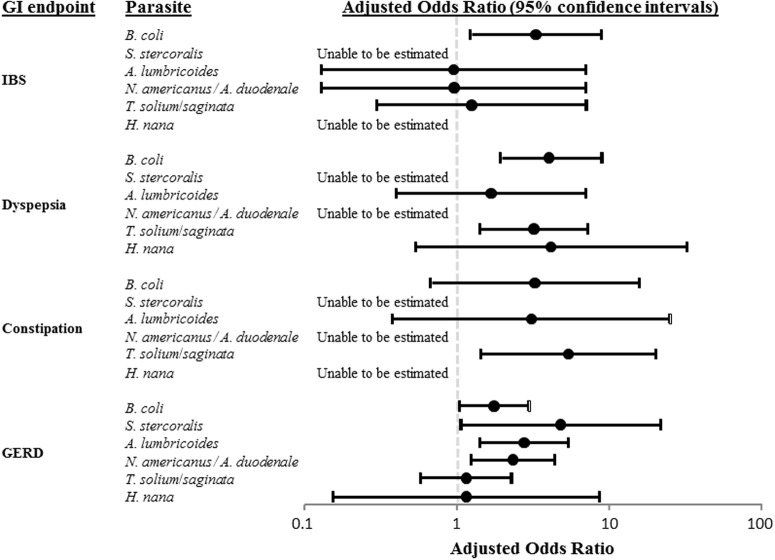
Pathogen-specific adjusted odds ratios (aORs) of irritable bowel syndrome (IBS), functional dyspepsia, functional constipation, and gastroesophageal reflux disease (GERD).

As shown in **Figure 1**, parasite-specific findings were outcome specific with *B. coli* associated with an increased risk of IBS (aOR of 3.22), *B. coli* and *Taenia* spp. with an increased risk of functional dyspepsia (aOR of 4.01 and 3.20, respectively), *Taenia* spp. with an increased risk of functional constipation (aOR or 5.32) and *B. coli*, *S. stercoralis*, *A. lumbricoides*, and *Necator americanus/Ancylostoma duodenale* with an increased risk of GERD (aOR of 1.73, 4.76, 2.74, and 2.32, respectively).

Females showed a statistically significant increased aOR for IBS (3.31) and non-significant increased aOR for all other outcomes. Race was not significantly associated with any of the outcomes of interest in univariate analyses. In multivariate analyses, military rank was significantly associated with functional constipation and GERD with Sr. enlisted personnel having a higher risk compared to all other active component ranks (aOR of 4.23 and 1.87, respectively). Neither prior IGE-related nor psychosocial-related medical encounters were significantly associated with an increased risk of any of the assessed endpoints. Deployment was not associated with any of the chronic-GI endpoints with comparable rates of deployment among those with and without the endpoints of interest (50–60%) (data not shown).

## Discussion

This study demonstrated an increased incidence for several chronic gastrointestinal disorders after infection with *Balantidium coli, Ascaris lumbricoides, N. americanus* and *A. duodenale,* and *Taenia solium* and *Taenia saginata* after controlling for known and potential confounders. To our knowledge, this is the first study to examine the risk of chronic gastrointestinal disorders after infection from these parasites. Although our study was limited to a primarily healthy, young, military cohort, a large proportion of the world’s population is known to be infected or at risk of infection to one or more of these parasites. Consequently this study provides observations which may need to be considered in evaluating the effect of these infections among populations where exposure rates are more common and high rates of environmental enteropathy are also notable (Arndt et al., 2016).

Our study found a significantly increased risk of IBS, functional dyspepsia, and GERD (borderline significant) after infection with *B. coli*. Wensaas et al. (2012), conducted a prospective cohort study of 2,500 subjects treated for giardiasis after infection from a contaminated water source. IBS was prevalent in 46.1% of exposed patients after 3 years of follow-up with an adjusted relative risk (aRR) of 3.4 (95%CI 2.9, 3.8) and CFS was prevalent in 46.1% of exposed patients after the same follow-up period with an aRR of 4.0 (95%CI 3.5, 4.5) (Wensaas et al., 2012). Other studies have reported higher prevalence of current infection with protozoan parasites such as *Blastocystis hominis, Dientamoeba fragilis,* and *Giardia lamblia* in patients diagnosed IBS compared to controls indicating that some patients with IBS may have untreated protozoan infections rather than PI-IBS (Nourrisson et al., 2014; Schwille-Kiuntke et al., 2015a). However, studies have also failed to identify associations, such as the Morgan et al. (2012) study of 163 Nicaraguan patients (endemic infection) diagnosed with IBS from a standardized Rome II criteria questionnaire and 194 healthy controls which did not find a statistically different carriage rate between the two groups for *B. hominis, G. lamblia, Entamoeba coli, E. histolytica, E. dispar, Iodamoeba butschlii,* or *Endolimax nana.* Patient population may be important as Krogsgaard et al. (2015) describes a case-control study of 124 IBS cases and 204 controls in Denmark in which a greater proportion of *D. fragilis* or *B. hominis* was identified in fecal samples from controls than cases (50% vs 36%, *p* = 0.01).

This is the first study describing an increased risk of chronic GI disorders following infection with several helminths. However, an increasing number of studies have highlighted the negative association between parasitic infections and allergic symptoms, though the data are conflicting. As highlighted in a recent review, while preclinical animal models point toward a negative causal association, clinical trials with helminths have been less consistent (Cruz et al., 2017). However, clinical studies to date have varied greatly in the disease and the parasite being assessed. Despite the variability, these studies highlight a complex relationship between host and parasite that likely involves the microbiome, immune response, nutrition, and other factors warranting additional study.

Proposed mechanisms for chronic sequelae following protozoan parasite infection include a complex interacting network of psychological, physiological, and microbiotic mechanisms triggered by the initial infection than may persist despite proper treatment (Gwee et al., 1999; Dizdar et al., 2007; Deising et al., 2013; Engsbro et al., 2014; Nourrisson et al., 2014). In a systematic review of animals models of PI-IBS for bacterial, parasitic, and chemical exposure, human IBS-like pathology was demonstrated with *Trichinella spiralis, Nippostrongylus brasiliensis,* and *Cryptosporidium parvum* infection (Qin et al., 2011). Gastrointestinal changes observed consisted of visceral hypersensitivity, altered gut motility and secretion, and mast cell hyperplasia. Motomura et al. (2010) examined chronic infection of mice with *Trichinella muris* to determine its effect on GI inflammation and motility. They found that low-grade colonic inflammation, altered microbiota flora colonization, and decreased smooth-muscle contractility persisted after eradication of the parasite which resembled changes seen in cases of IBS for these models. In another animal model, Hand et al. (2012) demonstrated that immune tolerance to commensal gut bacteria was lost after an acute GI infection with *Toxoplasma gondii* which could lead to a chronic pro-inflammatory state.

Beyond the associations with particular enteric parasite exposures, the associations of gender and military-specific covariates, we were unable to document an increased risk for any of the outcomes associated with a history of IGE related-disease, psychiatric disorders, or prior operational deployment; in contrast to other studies (Marshall et al., 2006; Spiller and Garsed, 2009; Porter et al., 2012). For example, several studies have reported the association of psychological disorders such as depression, anxiety, stressful life events, history of abuse, and personality disorders with functional bowel disorders (Longstreth et al., 2001; Drossman, 2006; Thabane et al., 2007; Porter et al., 2011a). Our lack of significant findings may be attributed to the smaller sample sizes, or could be population specific. Previous studies have reported a protective effect of prior operational deployment for the outcomes studied and have hypothesized a healthy worker effect (Curry et al., 2010; Porter et al., 2012). Deployed personnel tend to be younger, male, have little to no history of current or past chronic disease, and may be also less likely to report episodes of acute IGE or functional bowel disorders due to the potential impact a diagnosis may have operations or on their short- or long-term career intentions with the military.

This study had inherent limitations that could impact our effect estimates and generalizability. Caution is advised in the interpretation of our nearly universally positive results for a parasitic infection and its association with a subsequent increased rate of development of a FGID. Diagnostic bias may be introduced by patients who present with symptoms consistent with a FGID as they are likely to receive a robust diagnostic workup to rule out a potentially more serious organic cause. As part of this work-up, a parasitic infection may be identified which may or may not be causing the FGID symptoms. Additionally, the symptoms may be due to an active parasite infection (untreated or poorly treated) rather than a chronic change in the GI tract induced by an acute episode of enteric parasitosis previously treated and resolved.

Selection bias may have also impacted observed effects. Active component military personnel, in general, have easy and free access to medical services which may lead to increased medical seeking-behavior, elevated prevalence compared to the general population, and over diagnosis or misdiagnosis. However, young, male, active-duty personnel are also often less likely to seek medical care for clinical symptoms associated with an acute parasitic infection as well as chronic functional GI disorders due to time constraints, operational contingencies, and the potential impact on their short- and long-term careers. Importantly, we did not have data on antibiotic use to control for their potential confounding effects.

In summary, we observed a significantly increased risk of developing several chronic GI disorders after infection with a variety of enteric parasites. Increased risks were most consistently observed after infection with *B. coli*. The prevalence of functional bowel diseases both in the developed and developing world are expected to increase over the coming decades with increased awareness of these conditions both in providers and individuals as well as increased standardization of diagnostic criteria. As the pathogens investigated in our study represent a significant contribution to episodes of enteric parasite infection, especially in the developing world, efforts to understand the potential chronic effects of FGID are needed. In addition to further epidemiological research, we also recommend that causal mechanisms of FGID due to parasitic enteric infection be expanded from a primary focus on protozoan parasites. Utilization of proteomic, microbiomic, and other clinical investigation tools could be applied to both strengthen our understanding of the potential associations, as well as help identify mitigation and prevention strategies.

## Author Contributions

Two authors conceived of the research study (CP and MR), two performed the analyses (JB and CP), and all three (JB, MR, and CP) reviewed the study results and actively wrote and reviewed the final paper.

## Disclaimer

The views expressed in this article are those of the author and do not necessarily reflect the official policy or position of the Department of the Navy, Department of Defense, nor the U.S. Government. This is a U.S. Government work.

## Conflict of Interest Statement

The authors declare that the research was conducted in the absence of any commercial or financial relationships that could be construed as a potential conflict of interest. The handling Editor declared a past co-authorship with the authors MR and CP.
